# Bayesian state-space modelling of stock markets in G7 countries During the COVID-19 Pandemic

**DOI:** 10.1016/j.heliyon.2024.e39446

**Published:** 2024-10-16

**Authors:** Oluwadare O. Ojo

**Affiliations:** Department of Statistics, Federal University of Technology, Akure, Nigeria

**Keywords:** Bayesian, COVID-19, G7 countries, State-space modelling, Stock markets

## Abstract

This work examines the impact of Coronavirus disease (COVID-19) on the stock market of Group of Seven (G7) countries during the first wave of COVID-19 using daily data from March, 1st of 2020 to December, 31st of 2020. Focussing on such period, a Bayesian Structural Time Series Model (BSTSM) was used to capture the effects of first wave of COVID-19 on the stock market performance of these G7 countries by employing a Markov Chain Monte Carlo (MCMC) method. We considered an Autoregressive (AR) model with time-varying parameters and a local linear trend model to know if the stock price of these countries during the period of the first wave of COVID-19 is changing over time. There was a stochastic trend in stock prices of G7 countries during the period of the first wave of COVID-19 while the AR process itself was also changing over time. The stock market of the USA followed by Japan performed better than other G7 countries during the first phase of the COVID-19 pandemic while the stock market of France was affected during the COVID-19 pandemic.

## 1 Introduction

Since the detection and announcement of Coronavirus (SAR-COV-2) in the city of Wuhan, China in December 2019, the virus has become popular throughout the world. The disease was declared as a pandemic by the World Health Organization (WHO) on the 11th of March 2020.^1^ This pandemic has caused so many effects on the economy globally; 80 % of the international workforce has closed down their workplace; leading to the worst recession, of about 0.3 % deep compared to the great depression [1].

The Group of Seven (G7) is an organization that comprised of the world's seven largest advanced economies. These countries are; Canada, France, Germany, Italy, Japan, the United Kingdom (UK), and the United States of America (USA). This organization was established in the early 60s and its primary aim is to allow the world leaders of the seven countries to promote global change. As of today, the G7 countries are known as the seven wealthiest and most advanced countries in the world. Also, it was reported that G7 countries represent 40 % of global GDP and 10 % of the world's population [2].

The G7 countries have been seriously affected by this pandemic and several types of research have been carried out on the COVID-19 pandemic across the countries about their economies by different researchers (see Just and Echaust (2020), Hossain [3], Brown et al. [4], Knitkar [5], Narayan et al. [6], Yousaf and Ali [7] for more details). Few types of research on G7 countries about COVID-19 are not so much. However, the few researches on the COVID-19 pandemic in G7 countries are [8] and Izzeldin et al. [9]. Effects of the pandemic on stock markets in G7 countries was examined by [8] employing the Fourier-Shin cointegration test on daily data from January 15th, 2020 to April 24th, 2020. It was discovered that the COVID-19 pandemic has a negative effect on all the stock markets. Furthermore, the pandemic affected the Financial Times Stock Exchange Milano Indice de Borsa (FTSE MIB) stock of Italy mostly while Nikkei 500 stock of Japan was least affected. The most recent paper, Izzeldin et al. [9] investigated the impact of COVID-19 on stock markets across all G7 countries and their business sectors with a smooth heterogeneous autoregressive (HAR) model. This HAR model was built on the property of summation of short memory models that can give rise to hyperbolic decay pattern (see Ref. [10,11] for more discussion). Their results show a non-linear transition to crisis regime for all the G7 countries and sectors considered while the US and UK are the most affected countries with the highest heterogeneity in their business sectors response.

During the pandemic, several scholars have carried out research on the stock market prices of various countries. He et al. [12] examined both the spill-over and direct effect on COIVD-19 on stock markets in seven different countries namely; China, Italy, South Korea, France, Spain, Germany, Japan, and the USA with parametric (*t*-test) and non-parametric (Mann-Whitney test). They found out that COVID-19 has a negative effect and short term on stock markets. Also, a Structural Vector Autoregressive (SVAR) was employed by Yilmazkuday [13] to investigate the effects of the COVID-19 pandemic on the USA stock market. It was discovered that there is a 1 % of cumulative decline in their daily stock.

State-space models are specialized time series models used in handling time series data and modelling of a variety of behaviours. It was introduced by Kalman [14] followed by [15]. The model was primarily meant for use in aerospace-related research, for space tracking setting. However, its first use was demonstrated in modelling of economic data by Harrison and Stevens [16] followed by works of Harvey and Todd [17] & Kitagawa and Gersch [18]. Two principles that guide the usage of state-space model are: one, there is a latent process referred to as the state process and this state process follows a Markov process that is independent of one another. Two, observations are independent given the states; this simply means that dependence among the observations is generated by states (Kalma and Bucy, 1960).

State-space has become attractive recently in Bayesian modelling. It is flexible especially with the use of hierarchal prior, and this makes both the interpretation and computational methods similar in spirit to other models. A recent paper on the State-space model in the Bayesian framework is the work of Almarashi and Khan [19] while it has also been considered for modelling COVID-19 cases. The short time impact of COVID-19 on stock market performance in thirteen African countries was considered in Takyi and Bentum-Ennin [20] using the Bayesian state-space model in which the posterior estimates show that stock markets performance in African countries considered were reduced significantly. Also, Saini and Mittal [21] examined and compared the forecasting ability of both Autoregressive Moving Average (ARMA) and Stochastic Volatility (SV) models in state space and Kalman filter forms for the Indian stock market. It was revealed that forecast error was in favour of the SV model.

Studying the effects of first wave of COVID-19 pandemic on stock markets is considered important, as the COVID-19 outbreak was an unprecedented extraordinary public health challenge that brought hardships on people and economy. First wave of COVID-19 pandemic offers a unique opportunity to check the effects on stock markets so that government and policy makers can use it as reference point in future. It will also help the stock markets and market agents of G7 countries to react to exogenous events such as social unrest, natural disasters and political upheavals in the future.

For this reason, this present work therefore investigates the effects of first wave of COVID-19 pandemic on stock markets of G7 countries. Many countries are looking unto G7 countries by using their economies as a benchmark for their own countries, hence G7 countries stock markets need to be examined during the first wave of COVID-19 pandemic especially for recovery purposes. To capture the effect of the first wave of COVID-19 pandemic on the stock market in G7 countries, the Bayesian state-space model that encompasses Structural Time Series Model (STSM) will be utilized. The AR model with time-varying parameters and local linear trend models will be considered to know if the stock markets of the G7 countries during the period of the first wave of COVID-19 is changing over time.

The rest of this paper is organized as follows. Section 2 presents the method used in this study. Section 3 entails the presentation of data while Section 4 presents and discusses the results from the analysis. Section 5 renders the concluding remarks.

## 2 Material and methods

The work looks at the impact of COVID-19 on stock prices in G7 countries. The Bayesian Structural State Space Volatility (SSSV) will be used and is given as:

(1) $$y_{t}=Z_{t}\;\alpha_{t}+\varepsilon_{t}\text{,}$$

$$\varepsilon_{t}∼\;N\left(0,h^{-1}\right)$$

and

(2) $$\alpha_{t+1}=T_{t}\;\alpha_{t}+u_{t}$$

where.

$\alpha_{t}$ is P × 1

$\varepsilon_{t}$∼ N(0, ${\;h}^{-1}$)

$u_{t}$∼ N(0, ${\;H}^{-1}$)While $\varepsilon_{t}$ and $u_{t}$ are independent of one another.

In order to evaluate the impact of COVID-19 on the performance of stock markets in G7 countries, we use a simplified local level model with trend that evolves over time, hence we have:

(3) $$y_{t}=\mu_{t}+\varepsilon_{t}$$

(4) $$\mu_{t+1}=\mu_{t}+\tau_{t}+\omega_{t}$$

and

(5) $$\begin{array}{c} \tau_{t+1}=\tau_{t}+v_{t}\\ t=1,\ldots ,T \end{array}$$

where $y_{t}$ is the stock market index for each of the G7 countries, $\varepsilon_{t}∼\;N\left(0,\sigma_{\varepsilon }^{2}\right)$, $\tau_{t}∼\;N\left(0,\sigma_{\tau }^{2}\right)$, $v_{t}∼\;N\left(0,\sigma_{v}^{2}\right)$, all are independent of one another. Also, $\mu_{t}$ is the independent that changes with time and P×1 vector of P- state equations. $\tau_{t}$ is the component that allows linear trend.

To carry out a Bayesian inference on the model given in (3), (4), and (5) following DeJong and Shephard [22], Koop [23], Koop et al. [24], and more recently Chen and Strachan [25], we will use a suitable prior distribution in order to obtain a posterior distribution. Since $\mu_{t}$ and $\tau_{t}$ were well known, we simply follow the method of Chan and Jeliazkov [26] by stacking all states. Therefore, the state-space model in (1) can be written in a Normal linear regression model as:

(6) y=Xπ+ε

where.

ε∼ N (0, Σ)

y=${\left(y_{1},\ldots ,y_{T}\right)}^{\prime }$,

ε=${\left(\varepsilon_{1},\ldots ,\varepsilon_{T}\right)}^{\prime }$ with dimensions of (nT×1) and (nT×1) respectively, while

X = $\left[\begin{array}{ccc} \begin{array}{cc} x_{1} & 0 \end{array} & \cdots & 0\\ \begin{array}{cc} 0 & x_{2} \end{array} & \cdots & 0\\ \begin{array}{cc} \begin{array}{c} \vdots \\ 0 \end{array} & \begin{array}{c} \vdots \\ 0 \end{array} \end{array} & \cdots & \begin{array}{c} \vdots \\ x_{T} \end{array} \end{array}\right]$ and Σ = $\left[\begin{array}{ccc} \begin{array}{cc} \Sigma_{1} & 0 \end{array} & \cdots & 0\\ \begin{array}{cc} 0 & \Sigma_{2} \end{array} & \cdots & 0\\ \begin{array}{cc} \begin{array}{c} \vdots \\ 0 \end{array} & \begin{array}{c} \vdots \\ 0 \end{array} \end{array} & \cdots & \begin{array}{c} \vdots \\ \Sigma_{T} \end{array} \end{array}\right]$.

Thus, the likelihood function will have a standard form of a typical Normal regression model which is given as:

(7) P(y|π,h)=hT2(2π)T2{exp[−h2(y−xπ)′(y−xπ)]}

We assume an independent Normal-Gamma prior for π and h while a Wishart prior will be assumed for H. Thus, we write as:

(8) $$P\left(\pi ,h,H,\alpha_{1},{\ldots ,\alpha }_{T}\right)=P\left(\pi \right)\;P\left(h\right)\;P\left(H\right)\;P\left(\alpha_{1},{\ldots ,\alpha }_{T}|H\right)$$

where.

P (π) = $g_{N}$ ($\pi |\pi^{o},Q^{o}$)

${P\left(h\right)=g}_{G}$ ($h|{\left(S^{o}\right)}^{-2},v^{o}$)

$P\left(H\right)=g_{W}$ ($H|v_{H}^{o}$, $H^{o}$)

And

$$P\left(\alpha_{1},{\ldots ,\alpha }_{T}|H\right)=P\left(\alpha_{1}|H\right)\;P\left(\alpha_{2}|\alpha_{1},H\right).\;.\;.\;P\left(\alpha_{T}|\alpha_{T-1},H\right)$$

t=1,…,T−1

$${P\left(\alpha_{t+1}|\alpha_{t},H\right)=g}_{N}\left(\alpha_{t+1}|\;T_{t}\alpha_{t+1}\right)$$

$${P(\alpha_{1}|H)=g}_{N}\left(\alpha_{1}|\;0,H\right)$$

Combining (8) with likelihood function gives:

(9) $$P\left(\pi |y,\alpha_{1},\ldots ,\alpha_{T}\right),P\left(h|y,\alpha_{1},\ldots ,\alpha_{T}\right),and\;P\left(\alpha_{1},\ldots ,\alpha_{T}|y,\pi ,h,H\right)$$

The derivations of these conditional posteriors in (9) have been done in literature (see for [23] more details).

Hence, the posterior conditionals for parameters are given as:

(10) $$\pi |y\text{,}{h,\alpha }_{1},\ldots ,\alpha_{T}\;∼\;N\left(\pi^{*},Q^{*}\right)$$

(11) $$h|y\text{,}{\pi ,\alpha }_{1},\ldots ,\alpha_{T}\;∼\;G\left({\left(S^{-2}\right)}^{*},v^{*}\right)$$

(12) $$H|y\text{,}{\pi ,\alpha }_{1},\ldots ,\alpha_{T}\;∼\;W\left(v_{H}^{*},H^{o}\right)$$

where.

$Q^{*}$ = $\frac{1}{\left({\left(Q^{o}\right)}^{-1}+h\sum_{t=1}^{T}X_{t}^{\prime }X_{t}\right)}$.

$\pi^{*}$ = $Q^{*}\left[{\left(Q^{o}\right)}^{-1}\pi^{o}+h\sum_{t=1}^{T}X_{t}^{\prime }\left(y_{t}-Z_{t}\alpha_{t}\right)\right]$.

$v^{*}$ = T + $v^{o}$.

$$S^{*-2}=\frac{\sum_{t=1}^{T}{\left(y_{t}-X_{t}\pi -Z_{t}\alpha_{t}\right)}^{2}+{\left(vS^{2}\right)}^{0}}{v^{*}}$$

$$H^{*}=\frac{1}{\left[{\left(H^{0}\right)}^{-1}+\sum_{t=0}^{T-1}\left(\alpha_{t+1}-T_{t}\alpha_{t}\right){\left(\alpha_{t+1}-T_{t}\alpha_{t}\right)}^{\prime }\right]}$$

$$v_{H}^{*}=T+v_{H}^{o}$$

N.B: The symbols over the parameters with o and ∗ are prior and posterior while $g_{N}$, $g_{G}$, and $g_{W}$ are functions for Normal, Gamma, and Wishart distributions.

If we consider AR(p) model having a time varying parameters, we substitute the following vectors; $\alpha_{t}$ = $\left(\begin{array}{c} \alpha_{0t}\;\\ \alpha_{1t}\\ \begin{array}{c} \vdots \\ \alpha_{pt} \end{array} \end{array}\right)$, $u_{t}$ = $\left(\begin{array}{c} u_{0t}\;\\ u_{1t}\\ \begin{array}{c} \vdots \\ u_{pt} \end{array} \end{array}\right)$, $Z_{t}^{\prime }$ = $\left(\begin{array}{c} 1\;\\ y_{t-1}\\ \begin{array}{c} \vdots \\ y_{t-p} \end{array} \end{array}\right)$ into equations (1), (2) and by also setting $T_{t}$ = $I_{p+1}$ while $H^{-1}$ = $\left[\begin{array}{ccc} \begin{array}{cc} \frac{\lambda_{0}}{h} & 0 \end{array} & \cdots & 0\\ \begin{array}{cc} 0 & \frac{\lambda_{1}}{h} \end{array} & \cdots & 0\\ \begin{array}{cc} \begin{array}{c} \vdots \\ 0 \end{array} & \begin{array}{c} \vdots \\ 0 \end{array} \end{array} & \cdots & \begin{array}{c} \vdots \\ \frac{\lambda_{p}}{h} \end{array} \end{array}\right]$. Thus, we have AR(p) given as:

(13) $$y_{t}=\alpha_{0t}+\alpha_{1t}y_{t-1}+.\;.\;.+\alpha_{pt}y_{t-p}+\varepsilon_{t}$$

Where i=0,…,p.

And the state equation is also given as:

(14) $$\alpha_{it+1}=\alpha_{i,t}+u_{it}$$

Here, we assume that $\varepsilon_{t}$ ∼ N(0, $h^{-1}$) and $u_{it}$ ∼ N(0, λih) while both $\varepsilon_{t}$ and $u_{it}$ are independently and identically distributed. Since the errors in the state equation are uncorrelated, we only elicit prior for $\lambda_{i}$, i=0,…,p. Hence, the Wishart prior in equation (12) for this case will become:

(15) P(1λi)=gW(1λi|vio,1λio)

Just like the case of the local linear trend model as highlighted by West and Harrison [27] and Kim and Nelson [28], the conditional posterior for 1λi is given as:

(16) P(1λi|y,α1,…,αT)=gW(1λi|vio,1λio)

where.

$\lambda_{i}^{*}=\frac{h\sum_{t=0}^{T-1}\left(\alpha_{i,t+1}-\alpha_{it}\right){\left(\alpha_{i,t+1}-\alpha_{it}\right)}^{\prime }+v_{i}^{o}\;\lambda_{i}^{o}}{v_{i}^{*}}$.

And.

$v_{i}^{*}$ = T + $v_{i}^{o}$.

The Gibbs sampler and data augmentation methods will be used to sequentially draw from (10), (11), (12), (16) to obtain different estimates (see more details in Carter and Kohn [29], Geweke and Tanizaki [30], Choi [31], Whiteley et al. [[15], [32], [33], [34]]). We also set the prior hyper-parameters to be as $v_{i}^{o}=1,\lambda_{i}^{o}$ = 1, $S_{o}^{-2}$ = 1, $\pi^{o}$ = $\left(\begin{array}{c} 0\;\\ 0\\ \begin{array}{c} \vdots \\ 0 \end{array} \end{array}\right)$, $Q^{o}$ = $\left(\begin{array}{ccc} 0 & \cdots & 0\\ \vdots & \ddots & \vdots \\ 0 & \cdots & 0 \end{array}\right)$ for both local linear trend and AR(p) models.

## 3 Presentation of data

The data for this work are the daily stock market from 1st of March 2020 to 31st of December 2020 for seven countries namely Canada, France, Germany, Italy, Japan, UK, and USA during the first phase of COVID-19. Some stock markets will be used as proxies for all these countries to measure the performance of stock markets. They are S & P/TSX for Canada, CAC 40 for France, Deutscher Aktienindex for Germany, FTSE MIB for Italy, Nikkei for Japan, FTSE for UK, and Dow Jones indices for USA. The data were obtained from the database of the global financial portal at www.investing.com and www.gse.com. Table 1 shows the period when the pandemic was discovered in the G7 countries and their stock markets indices.

**Table 1:** Commencement dates for COVID-19 for G7-countries.

**Countries**	**Commencement date**	**Market Index**
Canada	January 27th, 2020	S & P/TSX
France	December 27th, 2019	CAC 40
Germany	January 27th, 2020	Deutscher Aktienindex
Italy	February 21st, 2020	FTSE MIB
Japan	January 16th, 2020	Nikkei
United Kingdom (UK)	January 31st, 2020	FTSE
United States of America (USA)	January, 20th, 2020	Dow Jones

Table 2 contains the summary of descriptive statistics. There is a decline in stock price for all the G7 countries. The USA has a mean of 26421.7706, seems to have performed well, followed by Japan with a mean of 22576.9740. Comparatively, Italy, Canada, Germany, and the UK performed averagely with 19205.9908, 15718.4172, 12096.3939, and 6025.6459, respectively while France was the least performing country out of all the G7countries with a mean of 4877.2668. The highest and lowest prices during the period are 30303.37 and 1228.49 for the USA and Canada, respectively. In terms of standard deviations, it was observed that France has the highest variation while the UK has the least variation.

**Table 2:** Descriptive statistics.

**Countries**	**Minimum**	**Maximum**	**Mean**	**SD**	**Skewness**	**kurtosis**
Canada	1228.49	17652.94	15718.4172	1313.89737	−0.982	0.871
France	3754.84	5609.15	4877.2668	39033.042	−0.227	0.051
Germany	8441.71	13667.25	12096.3939	1234.02921	−1.109	0.227
Italy	14894.44	22352.46	19205.9908	1677.6828	−0.266	−0.300
Japan	16552.83	27588.15	22576.9740	2466.11453	−0.135	−0.1251
UK	4993.89	6815.59	6025.6459	318.02926	−0.431	0.741
USA	18591.93	30303.37	26421.7706	2528.87641	−0.689	0.088

**Fig. 1:**
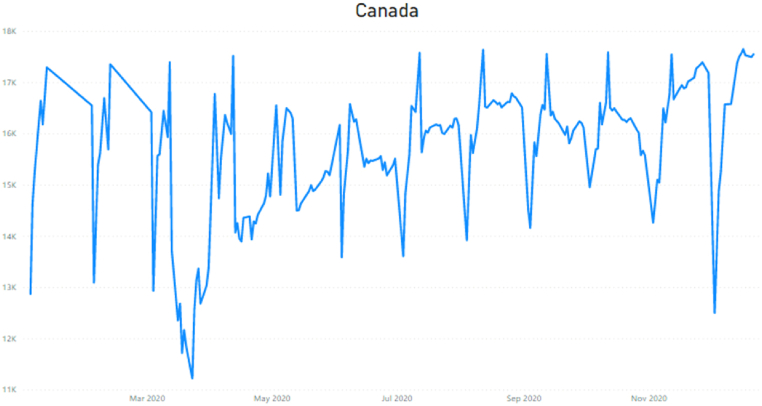
Plot of stock prices for Canada

**Fig. 2:**
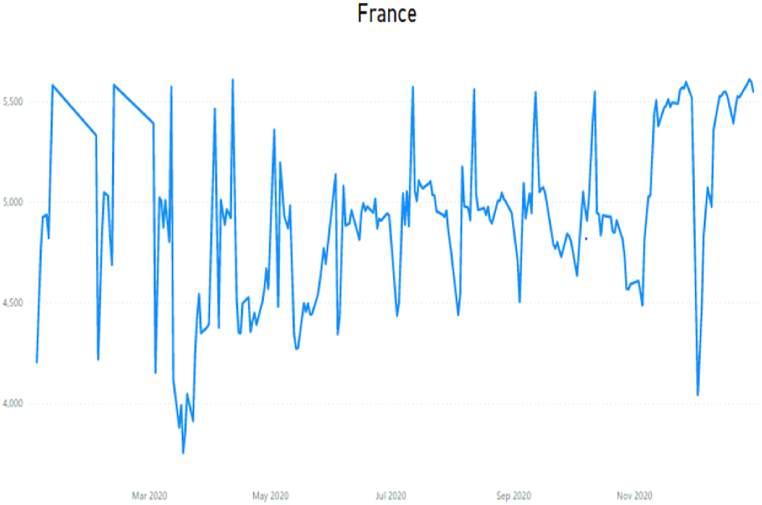
Plot of stock prices for France.

**Fig. 3:**
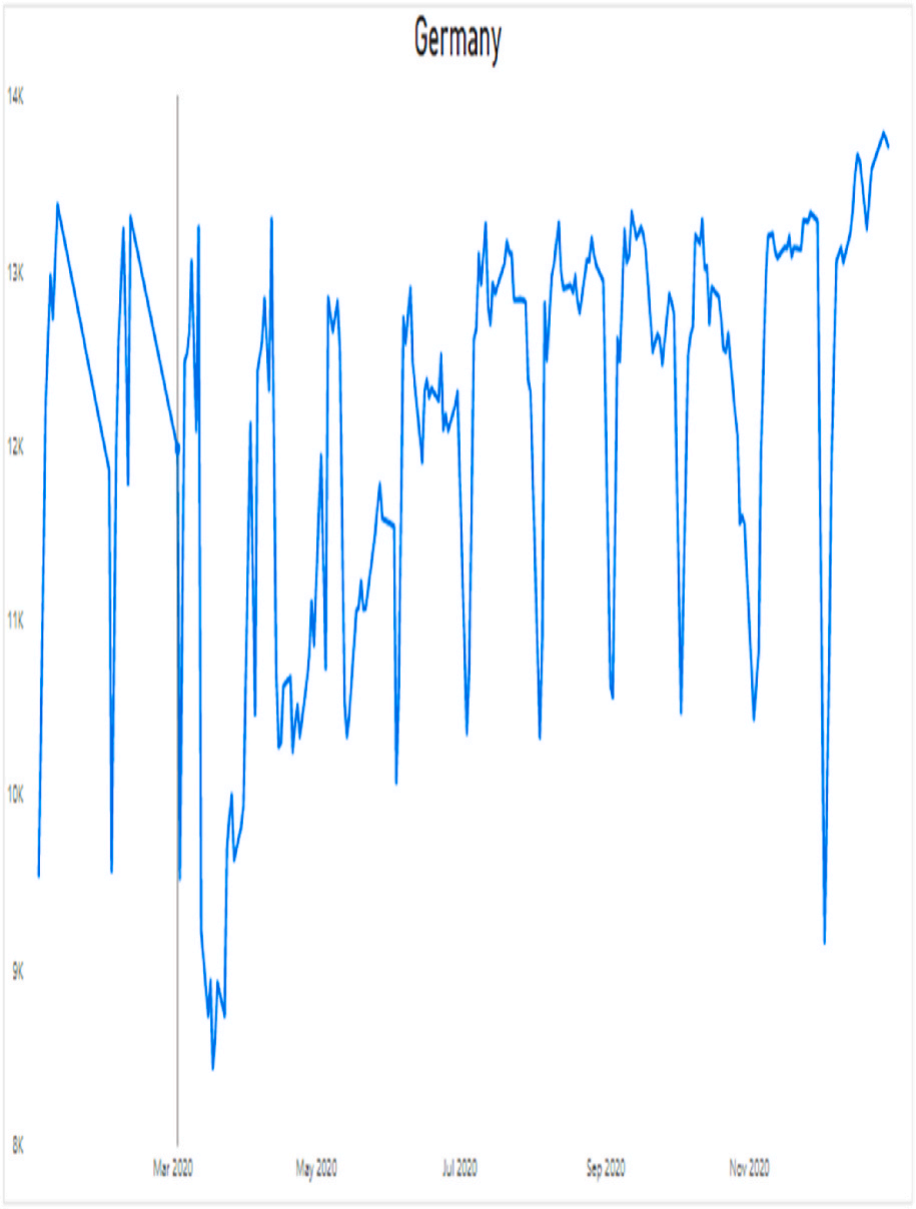
Plot of stock prices for Germany.

**Fig. 4:**
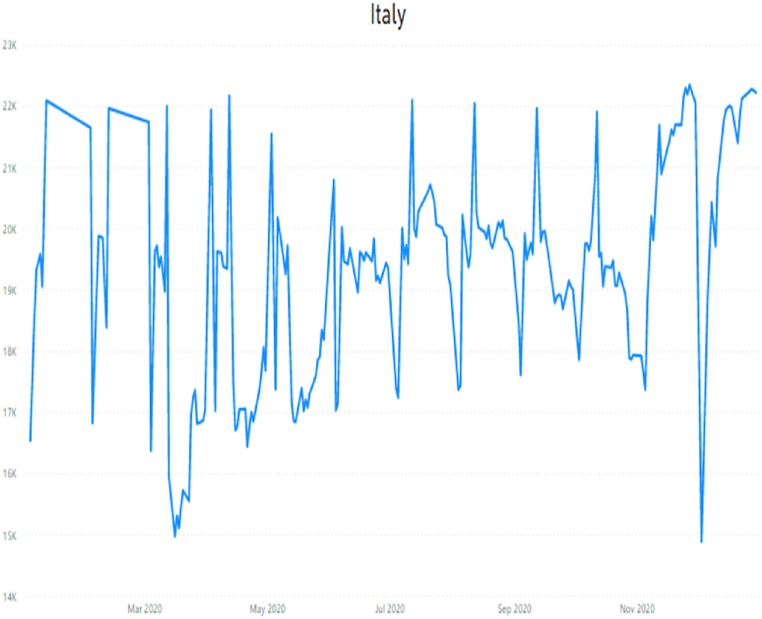
Plot of stock prices for Italy.

**Fig. 5:**
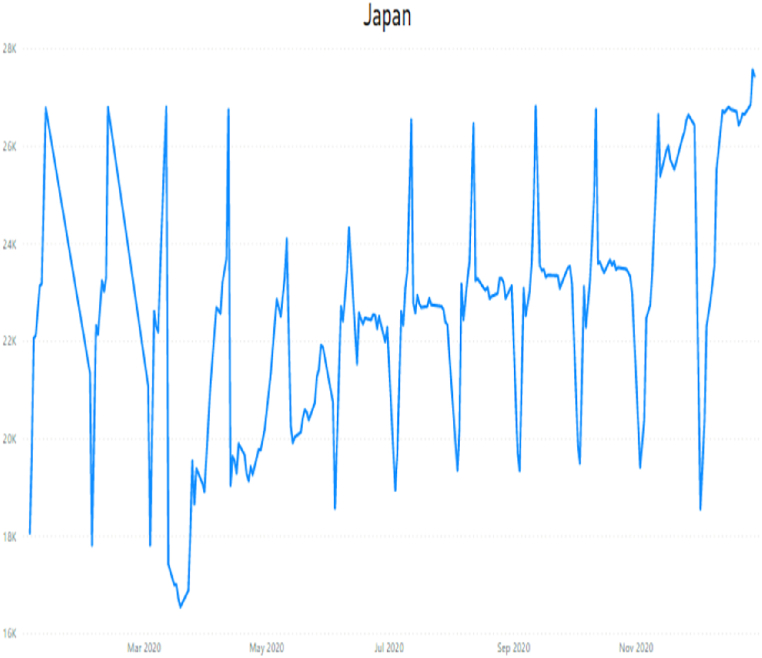
Plot of stock prices for Japan.

**Fig. 6:**
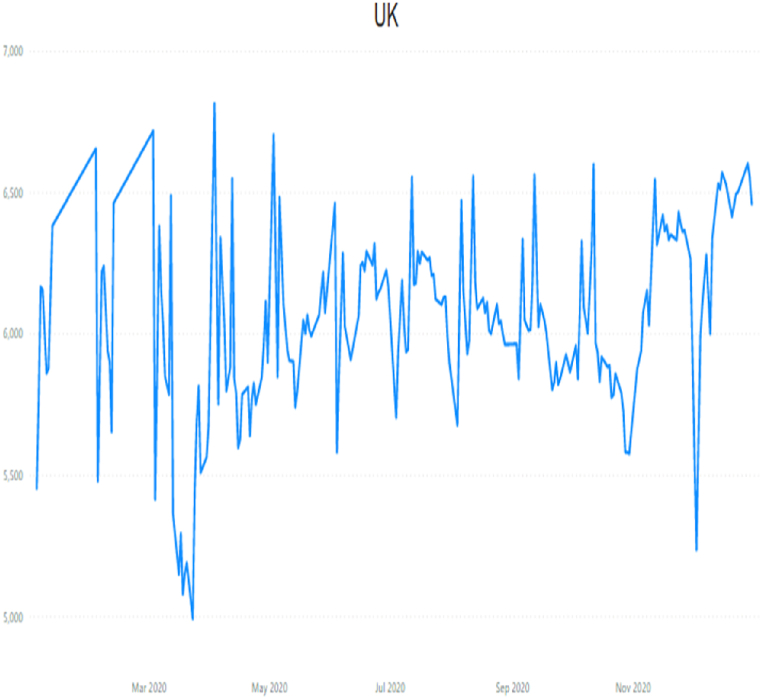
Plot of stock prices for UK.

**Fig. 7:**
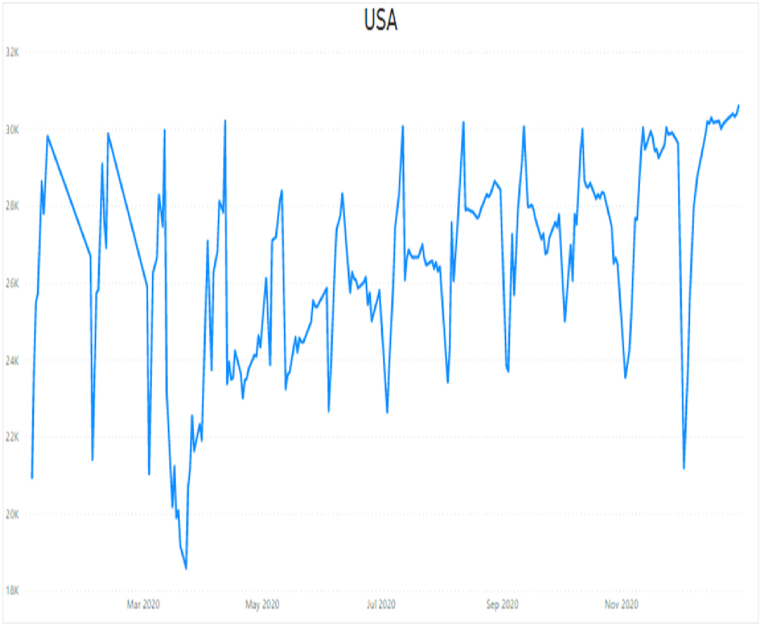
Plot of stock prices for the USA.

Figs. 1–7 as presented in the appendix also show the pattern and trend of stock prices in the G7 countries. Series of fluctuations were observed in all the countries between the periods of the first wave of COVID-19. These will help to further investigate the effects of the COVID-19 pandemic on stock markets of G7 countries during these periods, to know if the stock price during this period of the first wave of COVID-19 is changing over time.

## 4 Results and discussion

This Section contains the empirical results obtained from Bayesian analysis of the state-space model with AR (1) and local linear trend models in G7 countries during the first wave of the COVID-19 pandemic. Posterior estimates for intercept and AR (1) parameters were recorded for all the G7 countries in Table 3, Table 4. These include the mean, Standard Deviation (SD), Convergence diagnostic (CD), and Numerical Standard Error (NSE). Table 5 reports estimates (mean and credible intervals) for $\pi_{1}$ and $\pi_{2}$ using a local linear trend model.

**Table 3:** Results of Posterior estimates for AR model with time varying parameter $\lambda_{0}$.

**Countries**	**Mean**	**SD**	**CD**	**NSE**
Canada	1.0164	0.1991	0.5325	0.0020
France	1.0192	0.2007	0.7922	0.0020
Germany	1.0194	0.2001	0.1858	0.0020
Italy	1.0205	0.1997	0.1196	0.0020
Japan	1.0178	0.1987	−0.5208	0.0020
UK	1.0183	0.1986	−0.0060	0.0020
USA	0.0121	0.0095	−1.8190	0.0020

**Table 4:** Results of Posterior estimates for AR model with time varying parameter $\lambda_{1}$.

**Countries**	**Mean**	**SD**	**CD**	**NSE**
Canada	0.0119	0.0090	1.1915	0.0001
France	0.0094	0.0062	2.6182	0.0001
Germany	0.0123	0.0108	0.3870	0.0001
Italy	0.0141	0.0125	2.3570	0.0001
Japan	0.0103	0.0072	2.0053	0.0001
UK	0.0082	0.0052	−1.4924	0.0001
USA	1.0210	0.1972	0.5213	0.0001

**Table 5:** Results of Posterior estimates for local linear trend model on stock prices.

**Countries**	$\pi_{1}$		$\pi_{2}$	
	**Mean**	**CI**	**Mean**	**CI**
Canada	0.9302	(0.7896, 1.0682)	0.0324	(-0.1048, 0.1716)
Franc	0.9198	(0.8165, 1.0992)	−0.0050	(-0.1429, 0.1347)
Germany	0.9617	(0.8201, 1.1017)	0.0080	(-0.1453, 0.1279)
Italy	0.9377	(0.7953, 1.0739)	0.0292	(-0.1052, 0.1678)
Japan	1.0176	(0.0609, 0.0012)	0.0632	(-0.2000, 0.1000)
UK	0.9206	(0.7790, 1.0682)	0.0173	(-0.1192, 0.1527)
USA	1.0295	(0.9000, 1.2000)	0.0696	(0.2000, 0.1000)

Note: The values in the first and third columns are estimates for $\pi_{1}$ and $\pi_{2}$ while the second and fourth columns are the credible intervals (CI) for $\pi_{1}$ and $\pi_{2}$.

In both methods (AR with time-varying parameter and local linear trend), we make use of Gibbs sampler to fit the model. The simulator was run for 10, 000 replications, discarding the first 1000 as a burn-in period. $\lambda_{0}$ and $\lambda_{1}$ are drawn jointly from the posterior simulator to gain efficiency for time-varying parameter while $\pi_{1}$ and $\pi_{2}$ are obtained for local linear trend model for different stock markets.

**Fig. 8:**
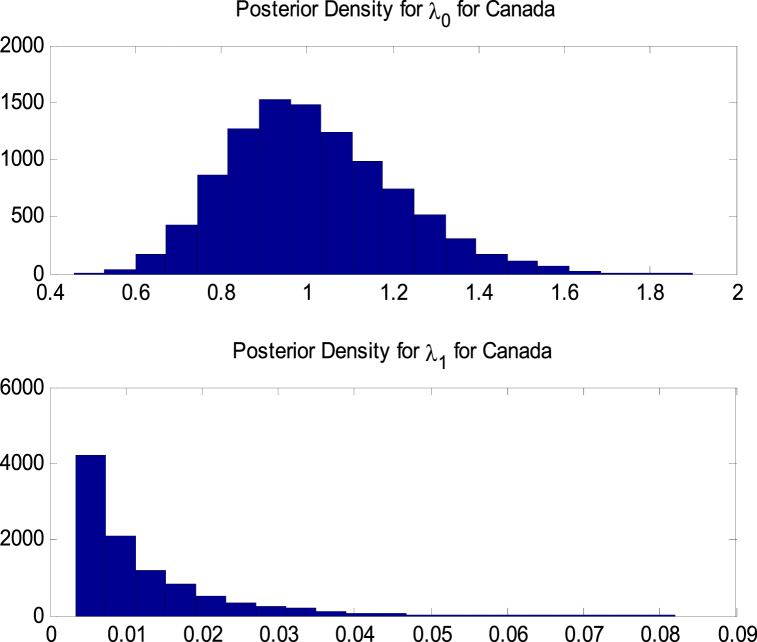
Histogram of Posterior density for Canada.

**Fig. 9:**
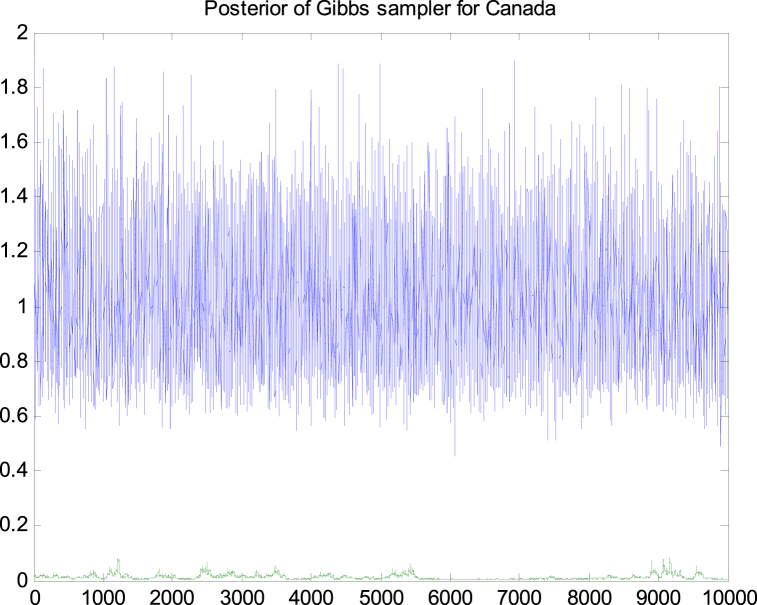
Plot of Posterior Gibbs sampler for Canada.

**Fig. 10:**
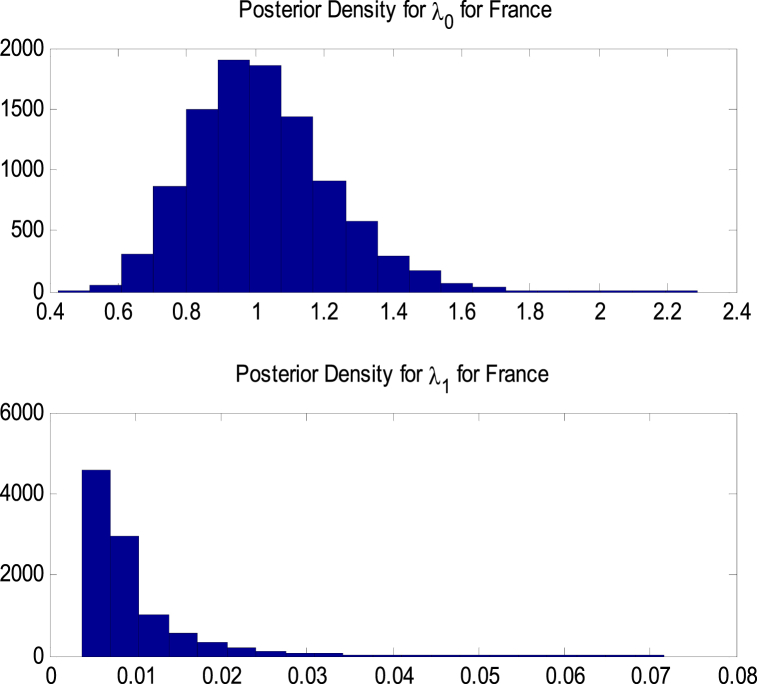
Histogram of Posterior density for France.

**Fig. 11:**
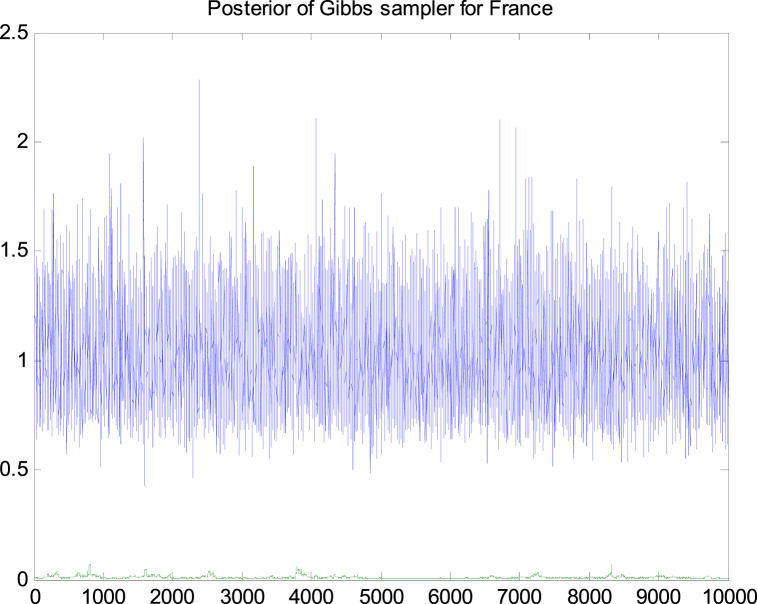
Plot of Posterior Gibbs sampler for France.

**Fig. 12:**
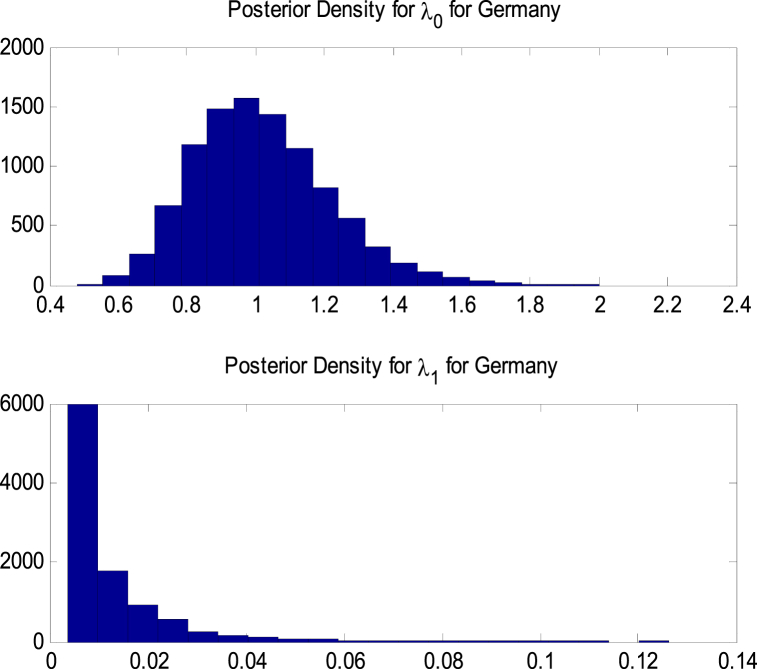
Histogram of Posterior density for Germany.

**Fig. 13:**
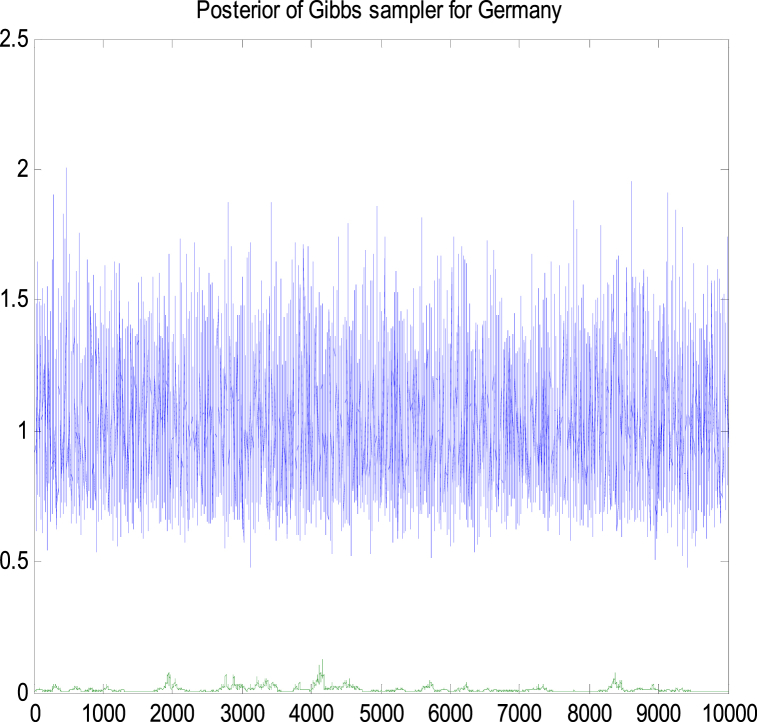
Plot of Posterior Gibbs sampler for Germany.

**Fig. 14:**
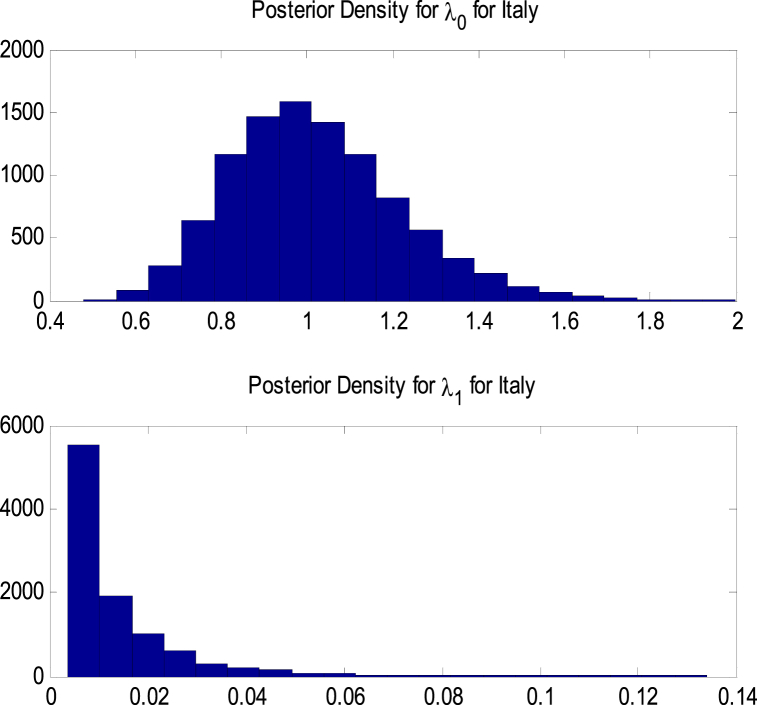
Histogram of Posterior density for Italy.

**Fig. 15:**
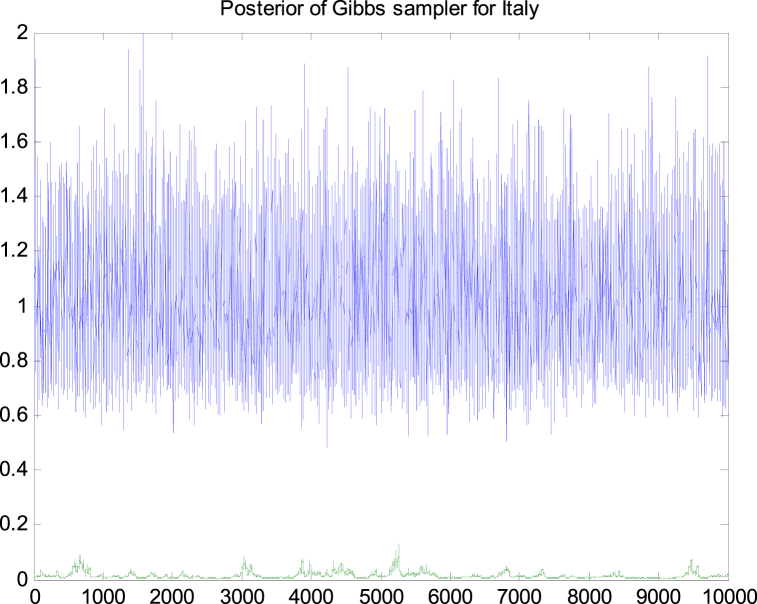
Plot of Posterior Gibbs sampler for Italy.

**Fig. 16:**
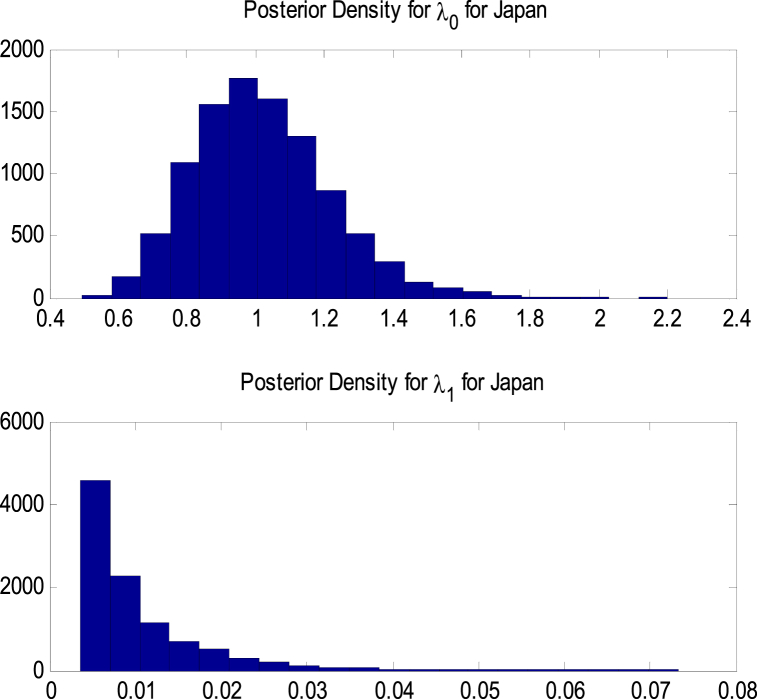
Histogram of Posterior density for Japan.

**Fig. 17:**
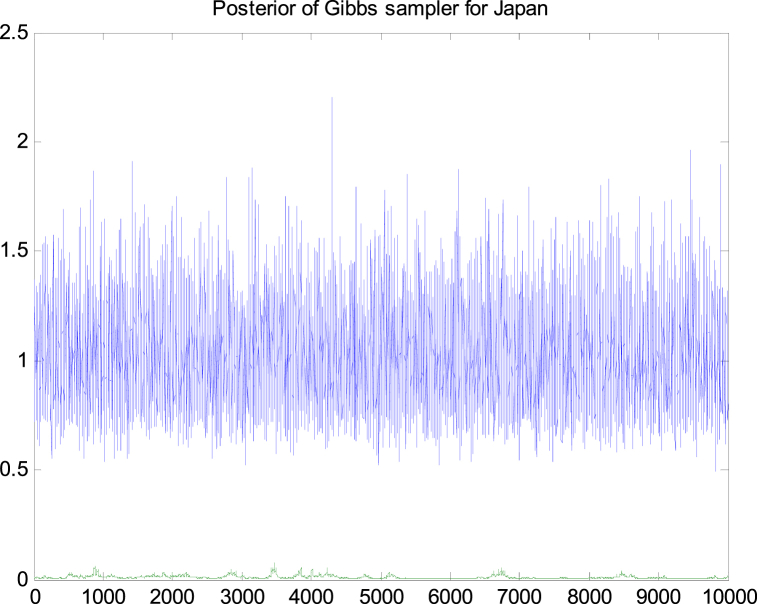
Plot of Posterior Gibbs sampler for Japan.

**Fig. 18:**
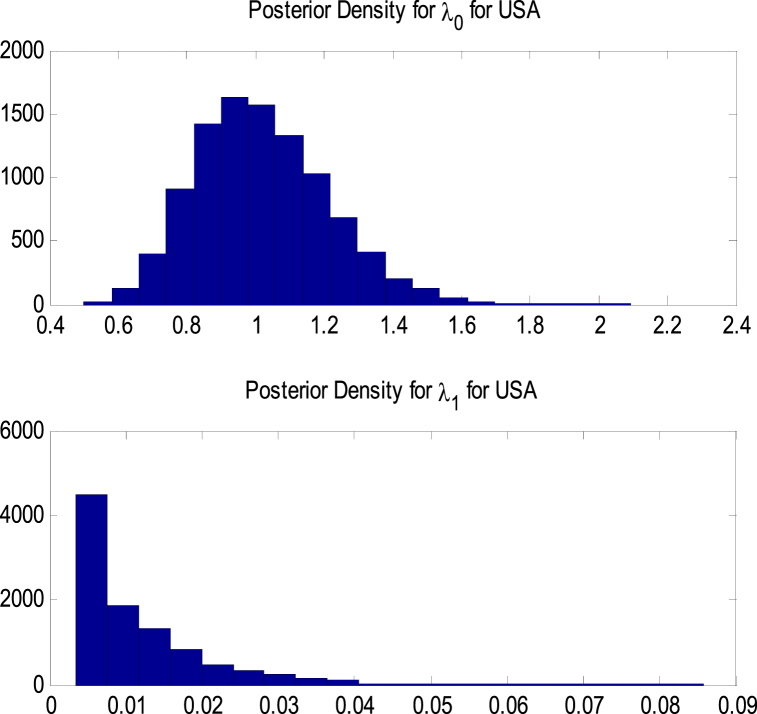
Histogram of Posterior density for the USA.

**Fig. 19:**
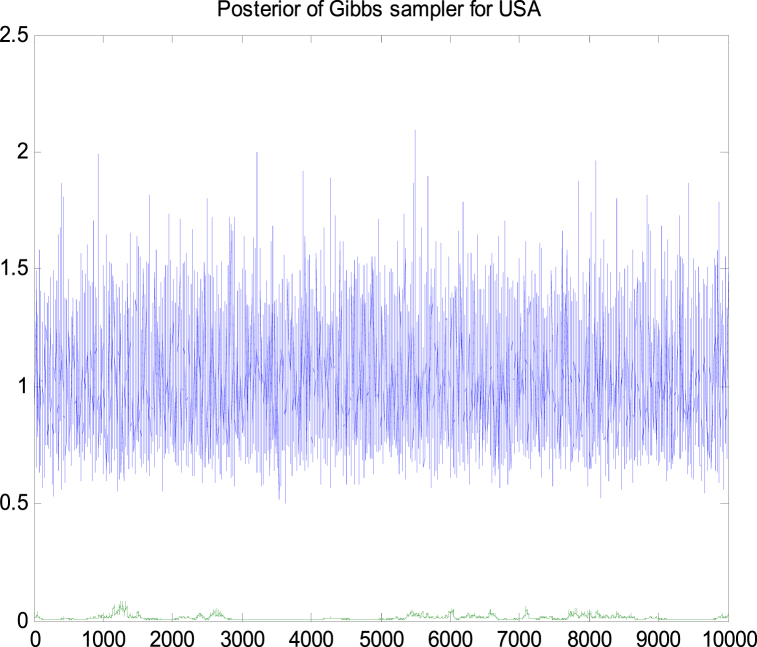
Plot of Posterior Gibbs sampler for the USA.

**Fig. 20:**
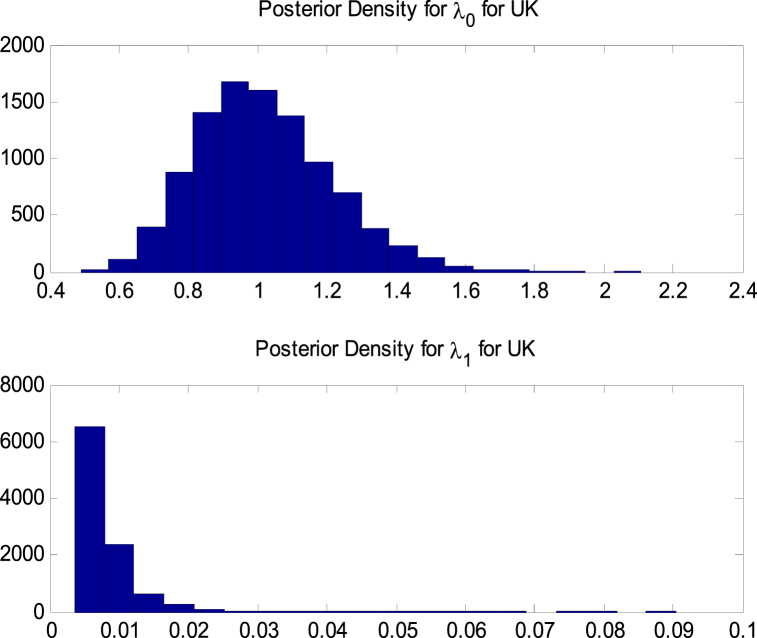
Histogram of Posterior density for the UK.

**Fig. 21:**
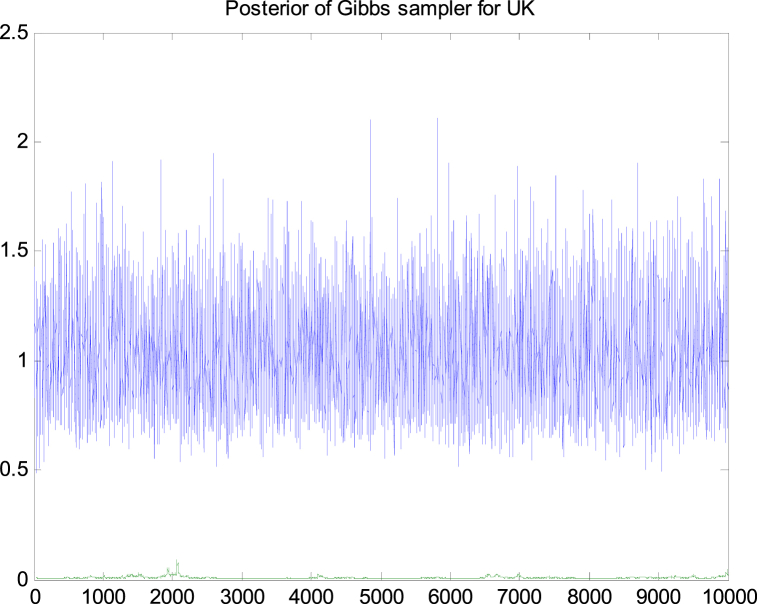
Plot of Gibbs sampler for the USA.

Table 3, Table 4, it was observed that the posterior means and standard deviation for both $\lambda_{0}$ and $\lambda_{1}$ show that amount of parameter variation occur in the intercept and AR(1) for all the G7 countries while there is also improvement in the stock prices for all the countries over time. The CD of both $\lambda_{0}$ and $\lambda_{1}$ for all the countries indicate that convergence of the Markov Chain Monte Carlo (MCMC) algorithms has occurred since CD is less than 1.96 in their absolute value except for France, Italy and Japan in the AR(1) parameter. There is also a noticeable stochastic trend in the performance of stock markets in G7 countries during the pandemic while the AR process itself was also changing over time. The Numerical Standard Error (NSE) for approximation of means for all the parameters ($\lambda_{0}$ and $\lambda_{1}$) show that the estimates obtained are quite accurate since they are all minimal. Figures 8–21 show the plots of histogram and Gibbs sampler of posterior densities. Figures 8, 10, 12, 14, 16, 18,and 20 are histogram for the AR model with time-varying parameters $\lambda_{0}$ and $\lambda_{1}$ while Figs. 9, 11, 13, 15, 17, 19, and 21 are plots of Gibbs sampler. The histogram has a shape that is quite skewed and also supported the findings. The Gibbs sampler also moves together in the plots, which means sufficient replications are taken.

In Table 5, the USA and Japan performed well at stock prices during the COVID-19 pandemic among the G7countries, their stock prices were averagely 1.0295 & 1.0176 and 0.0696 & 0.0632 for intercept and linear trend parameter, respectively. France seems to perform averagely low for both intercept and linear trend parameter with a mean of 0.9198 and −0.0050. Consequently, the Credible Intervals (CI) are tight on average and contain real stock prices for all the G7 countries during the COVID-19 pandemic.

## 5 Conclusion

There are numerous numbers of works on the impact of COVID-19 in developed countries. No much works have been carried out on stock markets of G7countries during the first wave of deadly COVID-19 pandemic especially with the use of Bayesian inference. The G7 countries comprise Canada, France, Germany, Italy, Japan, the United Kingdom (UK), and the United States of America (USA). These countries are not only the wealthiest but are the benchmarks other countries are using for their economies.

This work hereby focused on the impact of first wave of COVID-19 in G7 countries with the use of Bayesian inference that encompasses the Structural time Series model. The AR(p) model with time-varying parameters and local linear trend models were considered to know if the stock price of these countries during the period of the first wave of COVID-19 is changing over time. The stock market of the USA followed by Japan performed well than other G7 countries during the first phase of the COVID-19 pandemic while the stock market of France was affected during the COVID-19 pandemic. Hence, there was a stochastic trend in stock prices of G7 countries during the first wave of COVID-19 while the autoregressive process itself was also changing over time. It is also noteworthy that the evidence raised from this study is unique and can be used as a reference point on how G7 countries performed in the nearest future. Other time series models can be considered in the future works, such models should be able to estimate volatility of returns of stocks for G7 countries.

## Data availability

The data related to this manuscript can be obtained on request.

## Funding

This research did not receive any specific grant from funding agencies in the public, commercial, or not-for-profit sectors.

## Declaration of competing interest

The authors declare that they have no known competing financial interests or personal relationships that could have appeared to influence the work reported in this paper.
